# Abortion and Its Treatment: With Special Regard to the Injection of Warm Water

**Published:** 1880-05

**Authors:** J. Fletcher Horne

**Affiliations:** F. R. C. S. Edin.


### SELECTIONS.

---

### ABORTION AND ITS TREATMENT: WITH SPECIAL REGARD TO THE INJECTION OF WARM WATER.

BY J. FLETCHER HORNE, F. R. C. S. EDIN.

WARM-WATER therapeutics within the last two years have received considerable attention.

By reading a short paper by Dr. Atthill I was led to its use in abortion.

By the term abortion I imply the expulsion of the fœtus prior to the sixth month of pregnancy; I use it as synonymous with the popular expression—miscarriage.

Abortions of early pregnancy seldom need special treatment. The hemorrhage will usually be controlled by rest. Astringents may be tried if needful, or ergotine may be exhibited hypodermically.

In abortions of the eighth to the twelfth week we often have the ovum expelled with the unbroken membranes; if it be not, our aim must be to remove the ovum and its appendages, and thoroughly evacuate the uterus.

The uterine contractions suffice, in many instances, to burst the ovum and throw out the fœtus, and here the uterine action may cease; the pains do not recur as we could wish, the womb continues quiescent, the os almost closed, and the placenta and secundines remain. After a variable interval, from a few hours to as many days, the uterus will probably attempt to rid itself of its contents—often successfully.

Should there be no return of the pains, I think it desirable that immediate removal should be attempted. If the finger can be introduced, and pressure made outside the abdomen, the whole of the cavity of the uterus should be thoroughly explored and evacuated. If the os is closed it should be plugged with a tent, or Dr. Aveling's vaginal tampon will be found useful. The placenta, if retained, will usually become the seat of putrefactive change, as the following case will show:

Mrs. R., multipara, attended by midwife, who, having delivered a putrid fœtus and being unable to complete the removal of the placenta, asked me to see the patient. I carefully endeavored by the finger to remove the soft, pulpy, rotten mass to the greatest extent possible. No hemorrhage followed, but considerable pain. The fœtid discharge was kept down by Condy's fluid injection, and I several times removed further pieces. The case dragged on a weary existence for many months, and when the patient left the district she was far from well.

I now think that where a portion of the placenta remains, and you cannot reach it with the index finger, the use of the warm water steps in in preference to the use of the ovum forceps.

The tent or tampon will dilate the os and stimulate the uterine efforts. After its removal you will be able to scoop out the ovum and its surroundings.

In these cases I have frequently given ergot in its various forms, and have almost always felt disappointment at the result. Its action on the unstriped muscular fibre of the uterus, so well marked when the organ is fully distended, and the os fully dilated, is certainly not nearly so useful in the middle period of pregnancy. I find it produce tonic contraction of the os, and so act in a manner diametrically opposite to our desire. At the same time I would state, that I think it most desirable, after the whole of the contents of the uterus have been expelled, to stimulate the sympathetic system by the administration of ergot, so as to bring the arterial tree into a state of spasm, which closes the minute arteries, and prevents secondary hemorrhage.

It is a moot point whether absorption of the placenta occurs; if so, it explains the following case:—

Mrs. C. sent for me August 19th, 1879. Messenger said miscarriage. On arrival I found a fœtus of about twelve or fourteen weeks had come away. I carefully examined the clots, &c., for placenta. On vaginal examination found the small cord broken off almost in the closed os. On a little tension it again broke. There was no pain, no hemorrhage; nothing further came away, and in a few days, on my last visit, I found the patient in her shop.

In using the warm water I use an ordinary hand-basin, containing about three or four pints of the water at a temperature of 110° or 112°. I would here strongly recommend any of my readers who would use this plan not to use their clinical thermometer; if so, they will find that they will not be able to again shake down the index. I find it a safe limit to use water sufficiently warm that you can hold your hand in it without any degree of discomfort. The basin is placed close to the nates, and one of Higginson's enemas, with vaginal tube attached, is carried up with the fingers through the os uteri, the water is then to be gradually injected until complete contraction follows—usually one or two pints suffice.

I append three cases of abortion in which I have used the warm water with complete success. In one case of post-partum hemorrhage at term in which I used it, the warm water was not so successful.

CASE I.—Mrs. D., aged forty, sent for me on the night of April 7th, 1878. I subsequently learned that she had had nine children, and found that she had missed three menstrual periods, and ten days before had begun to be unwell; and this had continued without treatment till this afternoon, when a fœtus of about the eighteenth week of pregnancy came away. This was followed by profuse hemorrhage, which was succeeded by fainting and tossing of the arms about—symptoms so characteristic of the drain that had taken place. On my arrival I found her almost pulseless, blanched, the bed saturated with blood, vagina full of clots. By introducing the index finger through the partially dilated os uteri, and with external pressure I got away a portion of the miniature placenta, but the hemorrhage still continued. I then injected gradually about two pints of warm water, with the result of bringing into reach the rest of the placenta, and also producing immediate contraction of the uterus, my finger being expelled by the uterine action.

CASE II.—Mrs. B., a very delicate woman, aged about forty, pregnant of her seventh child, was delivered August 24th, 1879, at 7 P. M., of a fœtus of about the fifth month, which apparently had been dead some time. The placenta came away by external pressure on the uterus. Three hours after, her attendants having tilted her up, violent hemorrhage followed, which drained the woman almost to death's door. On my arrival I immediately injected warm water with success, the hemorrhage ceasing immediately.

CASE III.—Mrs. W., aged thirty-eight, pregnant of her twelfth child, aborted October 15th, 1879. A fœtus of about the third month came away about 2 P. M. At 9 P. M., on my seeing her,

I found she had been losing all day, had fainted several times, was blanched and pulseless. On my injection of warm water collapse followed at once, and the patient turned over as if about to die, but immediately rallied. Contraction of the uturus ensuing almost immediately, a dose of ergot was given; slight discharge continued all night. Nourishing food and tonics were subsequently given, and the patient soon became convalescent.

Physiologically this treatment brings out the nervi-motor power—the reflex action being stimulated by the warm douching and by the impression upon the internal uterine surface, and perhaps upon the uterine muscular fibres. By the administration of ergot after the abortion is completed, the direct spinal action is also stimulated, and no further hemorrhage takes place. —*Obst. Journal of Great Britain and Ireland.*

